# Clinicians' opinion on massage in the intensive care unit patients

**DOI:** 10.3389/fpain.2025.1452434

**Published:** 2025-04-09

**Authors:** Tomasz Zwoliński, Marta Jaskulak, Konrad Janicki, Bartłomiej Siek, Dominika Batycka-Stachnik, Bartosz Wilczyński, Dominika Szalewska, Kamila Gworys, Piotr Wąż

**Affiliations:** ^1^ Department of Health, University WSB Merito Gdańsk, Gdańsk, Poland; ^2^ Department of Immunobiology and Environmental Microbiology, Medical University of Gdańsk, Gdańsk, Poland; ^3^ Faculty of Health Sciences, Medical University of Gdańsk, Gdańsk, Poland; ^4^ Department of History and Philosophy of Medical Sciences, Medical University of Gdańsk, Gdańsk, Poland; ^5^ Clinical Department of Heart, Vascular and Transplant Surgery of St. John Paul II, Cracow Specialistic Hospital, Kraków, Poland; ^6^ Division of Rehabilitation Medicine, Faculty of Health Sciences, Medical University of Gdańsk, Gdańsk, Poland; ^7^ Department of Physical Rehabilitation Medicine, Medical University of Łódź, Łódź, Poland; ^8^ Department of Nuclear Medicine, Faculty of Health Sciences, Medical University of Gdańsk, Gdańsk, Poland

**Keywords:** anxiety, bowel movement, fear, intensive care unit, massage, pain, physical therapy, sleep

## Abstract

**Introduction:**

Physiotherapy in the Intensive Care Unit (ICU) is a common medical procedure involving mainly elements of mobilisation, electrotherapy and also, in various forms and to a lesser extent, elements of massage. Massage can positively influence the physical and psychological outcomes of the ICU) patients.

**Aim:**

The study aimed to assess the perception of physiotherapists (PTs), physicians (PHs), and registered nurses (RNs) working in ICU about the possibilities and safety of implementing massage in the process of rehabilitation of ICU patients.

**Methods:**

This multicentre survey was conducted in Poland in three ICUs (Gdansk, Koscierzyna, Krakow). A total of 135 people participated in the study. Of these, 25.9% (35/135) were PTs, 21.5% (29/135) were PHs, and 52.6% (71/135) were RNs. The questionnaires were distributed and collected online (directly by respondents to Google Form system) and in written form.

**Results:**

Most PTs—71% (25/35)—perceive massage as a beneficial and safe treatment while working with ICU patients. PHs 96% (28/29) and RNs 92% (65/71) also recommend using massage by physiotherapists to rehabilitate ICU patients. In the respondents' opinion, the possibilities for the use of massage by PHs and RNs are lower (between 20% and 55%).

**Conclusions:**

According to the surveyed clinicians working in the ICU, massage is a safe form of treatment, especially when performed by PTs and it could be a permanent element of rehabilitation among ICU patients, especially for reducing pain, anxiety and restlessness and also improving sleep quality and bowel movement.

## 1 Introduction

Massage is a structured therapeutic procedure applied manually on a patient's skin to induce soft tissue mobilization and is considered beneficial for improving a patient's treatment process (1, 2). It is well-known that patients in Intensive Care Units (ICUs) experience increased pain, sleep deprivation, and sensory overload (3–5). Consequences of these experiences may include prolonged mechanical ventilation, long hospital ward stays, posttraumatic stress, and later complications (4, 6). Pharmacological treatment of the above mentioned symptoms in the ICU is the primary form of care. Physiotherapy, implemented from the first days of a patient's ICU stay, is also an integral form of treatment. Recently, there has been a growing body of scientific evidence supporting the need for physiotherapy in the ICU, including massage. Massage may involve the manual manipulation of soft tissues, such as muscles and connective tissues, usually involving skin-to-skin contact, with the purpose of promoting relaxation, reducing pain, and enhancing overall well-being (7). It can be applied to the whole body or targeted to specific areas depending on the therapeutic goal (8). Importantly, structured therapeutic massage should be distinguished from simple comforting gestures like holding a patient's hand or gentle touch, which, while providing emotional support, lack the intentional techniques and therapeutic objectives that define massage (9). Despite this, it is still not *a priori*ty approach (10). Lack of knowledge and training, redundancy of hospital staff, and clinical guidelines that do not include this approach are some of the possible reasons why the implementation of massage in the treatment of ICU patients is at a low level (4, 10, 11).

It is worth noting that research carried out by Lindgren et al. has shown that touch massage influences the physical and psychological outcomes of ICU patients. It can have a beneficial effect on reducing heart rate, blood pressure, and respiratory rate. In addition, it is also able to reduce pain, anxiety, and stress levels (12). Moreover, Kutner et al. found that the use of simple touch should be worth considering in improving the quality of life of hospice patients (13). Furthermore, it is a well-known fact that nursing staff often use touch to bond and provide comfort to patients. The research of Henricson et al. confirmed the significant positive impact of touch on quality of life with emphasis on the emotional nature of ICU patients which was given by the nursing staff (14). Touch in itself, despite its proven beneficial effects (15, 16) remains something poorly captured. There is little clinical research addressing its relevance. Even physiotherapists, whose profession is largely based on working with the body (17), often treat touch intuitively, without thinking about its intention, without planning it in a way that, for example, movement exercises are planned. It is worth considering the importance of touch itself, which is an integral part of a massage, but also of physiotherapy as a medical profession. First, it is worth noting that simple touch alone can reduce pain and improve the psychological wellbeing of patients. In addition, it provides patients with a break from the stressful experiences of hospitalization, allows them to experience a moment of pleasure, and thus builds a feeling of hope, which is important in the recovery process (13–15, 17).

This study examines the feasibility and perceived benefits of massage as an intervention for ICU patients, focusing on the role of physiotherapists and the potential involvement of nurses and physicians. However, this thesis may seem to be controversial due to the overwork of hospital staff and the lack of training of nurses and physicians. Therefore, the question is whether and who could help patients in ICU by providing massage interventions.

The first purpose of the current research was to know the opinion of ICUs medical staff (physiotherapists, nurses, physicians) on the potential effects of massage performed by physiotherapists on the outcomes of patients treated in the ICU. Moreover, the questionnaire survey verifies the potential feasibility of performing massage also by nurses and physicians in the treatment's process of ICU patients. To check these questions the clinicians' opinion working in ICUs in Poland was examined referring to the possibilities of the application of massage in ICU patients.

## 2 Materials and methods

This study was a multicentre questionnaire survey conducted in three, located in the different cities, intensive care units in Poland. The survey was designed to allow respondents to complete the questionnaire both online and on paper. The survey aimed to verify the opinions of clinicians working in intensive care units on the feasibility of using massage as part of physiotherapy for ICU patients. The Independent Bioethics Committee for Research at the Medical University of Gdansk, after reviewing the preliminary draft of this survey, has stated in an official email response that the survey presented does not require official approval. In the Supplementary Appendix Table S6 are details of the methodology of this survey, for which the Checklist for Reporting Results of Internet E-Surveys (CHERRIES) tool was used (18).

### 2.1 Participants and data collection

All medical personnel, who participated in the presented survey, completed the questionnaire voluntarily and anonymously. Participants were invited in person or online by physiotherapists working in intensive care units, as well as coordinators of nursing, medical and physiotherapy staff. A questionnaire survey was invited from 266 participants. A total of 135 people eventually completed the survey. The questionnaire survey was carried out from January 2021 until June 2022 and was conducted at Anaesthesiology and Intensive Care Unit, University Clinic Centre in Gdansk, Poland; Specialised Hospital in Koscierzyna, Poland; St. John Paul II Hospital in Krakow, Poland. The questionnaires were distributed and collected both online (directly by respondents to Google Form system) and in written form by physiotherapists working in ICUs (KJ, TZ, DB-S). The written questionnaires were manually loaded into the Google Form system (KJ). Next, the number and type of the participants were downloaded from the Google Forms system directly (TZ). Then, the final data set was exported into Microsoft Excel spreadsheet and the detailed statistical analysis was performed to be presented in written and graphical forms in this paper (BS, PW, TZ).

### 2.2 Questionnaire

In order to explore the opinions of representatives of all groups working in the ICU, four questions were formulated regarding the usefulness of massage by physiotherapists in the treatment of ICU patients. Each of the following questions could be answered with one of the following: yes, no opinion, no. The questions were: (1) The use of massage by physiotherapists may improve the sleep quality of ICU patients. (2) The use of massage by physiotherapists may reduce the level of fear and anxiety in ICU patients. (3) The use of massage by physiotherapists may reduce pain in ICU patients. (4) The use of massage by physiotherapists may have a positive effect on the peristalsis of the large intestine and the prevention of constipation in ICU patients. In order to determine how likely it is that representatives of professions other than physiotherapists are also involved in massage in ICU patients, two questions were formulated, but linked to each professional group, hence the answers obtained include six versions of the questions. Also, for these questions one answer could be given: yes, no opinion, no. The questions were: (1) The use of massage by physiotherapists should be a permanent element of the ICU patients rehabilitation. (2) The use of massage by physiotherapists is safe for ICU patients. (3) Elements of massage used by ICU physicians, after appropriate training, could be a part of the basic rehabilitation procedures of ICU patients. (4) Elements of massage used by ICU physicians, after appropriate training, would be safe for ICU patients. (5) Elements of massage used by ICU nursing staff could be a part of the basic rehabilitation procedures of ICU patients. (6) Elements of massage used by ICU nursing staff would be safe for ICU patients. The questions in the first group were used to determine the extent to which the opinions expressed by ICU staff are consistent with the advantages of using massage on ICU patients as presented in the literature. The compilation of responses from both groups, in turn, made it possible to assess whether the knowledge and opinions in each professional group could translate into their willingness to provide massage to ICU patients (Supplementary Appendix).

### 2.3 Statistical methods

For qualitative variables, the primary statistic was the frequency of a specific category for the trait under study. Fisher's exact test for count data and the *χ*2 test were used to test the relationship between the categories of the two traits. Test of proportion was also used to analyse the collected data.

For each case in which statistical inference was applied and a significantly statistical result was obtained, the power of the test used was determined (pwr = 1-β, where β is the probability of making a Type II error).

A significance level of *α* = 0.05 was adopted.

Calculations were performed using functions and procedures available in the R project environment (19).

## 3 Results

The detailed characteristics of the 135 participants in terms of gender, workplace and occupation who took part in the survey are shown in Table 1.

**Table 1:** The detailed characteristics of the 135 participants.

Profession % (*n* = 135)	Gender		ICU in Gdansk	ICU in Koscierzyna	ICU in Krakow
	Women	Man			
	(*n* = 105)	(*n* = 30)	(*n* = 75)^A^	(*n* = 42)^S^	(*n* = 18)^J^
Physiotherapists (PTs)	21	14	6	22	7
26% (35/135)					
Physicians (PHs)	14	15	20	9	—
22% (29/135)					
Registered nurses (RNs)	70	1	49	11	11
52% (71/135)					

A = Anaesthesiology and Intensive Care Unit, University Clinic Centre in Gdansk, Poland.

S = Specialised Hospital in Koscierzyna, Poland.

J = St. John Paul II Hospital in Krakow, Poland.

In the case of ICU Gdansk, the “survey return” rates for physiotherapists, physicians and nurses are 60%, 44% and 71%, respectively. The result of the test of proportion in this case is statistically significant (*p*-value = 0.01723, pwr = 0.96). The calculated the “survey return” for the Koscierzyna ICU are 88%, 100% and 61%, respectively, and for the Krakow ICU are 35% and 16% (only the ratios for physiotherapists and nurses were determined for the Krakow ICU). The result of the test of proportion test for the Koscierzyna ICU are statistically significant (*p*-value = 0.03105, pwr = 0.61), and for the rates determined for the Krakow ICU are not statistically significant (*p*-value = 0.10850). The “survey return” rates were also determined for each professional group (separately) in the three studied ICUs (Gdansk, Koscierzyna and Krakow). For physiotherapists, the “survey return” rates were 60%, 88% and 35%, respectively, for physicians 44%, 100% (calculated for Gdansk and Koscierzyna ICUs only), and for nurses 71%, 61% and 16%. The tests of proportions used yielded significantly statistical results in each of the cases studied (*p*-value = 0.00090, pwr = 0.96; *p*-value = 0.00227 pwr = 0.94 and *p*-value < 0.00001, pwr = 1, respectively).

Firstly, the opinions of medical staff working in ICUs on the usefulness of massage by physiotherapists working in ICU to improve sleep quality and bowel movements and to reduce anxiety, restlessness and pain among ICU patients were checked. The detailed distribution of responses in each professional group is shown in Table 2. According to the majority of respondents, massage performed by physiotherapists working in the ICU could improve the sleep quality of ICU patients. An affirmative answer was given by 85.6% (30/35) PTs, 89.7% (26/29) PHs and 81.7% (58/71) RNs). Also, an overwhelming proportion of participants in the current study recommend the use of massage by physiotherapists to reduce anxiety and distress associated with ICU patients. This question was answered “yes” by 88.7% (30/35) PTs, 93% (27/29) PHs and 74.7% (53/71) RNs). When it came to reducing the level of pain experienced by ICU patients, too, massage by ICU physiotherapists was rated very positively by all participants in the present study. It is worth noting that 88.7% (30/35) of PTs; 93% (27/29) of PHs; 74.7% (53/71) of RNs) answered “yes” to this specific question. Moving on to the final question in this part of the survey, concerned the possibility of physiotherapists working in the ICU using massage to improve intestinal peristalsis in ICU patients. As in the previous questions, the vast majority of respondents answered this question positively, with 88.5% (31/35) PTs, 86.2% (25/29) PHs; 90.2% (64/71) RNs) replying “yes”.

**Table 2:** Opinions on the usefulness of massage by physiotherapists in the prevention and treatment of selected medical problems in ICU patients.

Question (summarised)	Responses		
	Yes	No opinion	No
	*n* (%)	*n* (%)	*n* (%)
In physiotherapists' opinions (*n* = 35) massage may			
Improve the sleep quality	30 (85,7%)	4 (11,4%)	1 (2.9%)
Reduce the level of fear and anxiety	31 (88.6%)	3 (8.6%)	1 (2.9%)
Reduce pain	33 (94.3%)	1 (2.9%)	1 (2.9%)
Have a positive effect on the peristalsis	31 (88.5%)	1 (2.9%)	3 (8.6%)
In physicians' opinions (*n* = 29) massage may			
Improve the sleep quality	26 (89.7%)	3 (10.3%)	—
Reduce the level of fear and anxiety	27 (93%)	1 (3.5%)	1 (3.5%)
Reduce pain	28 (96.5%)	1 (3,5%)	—
Have a positive effect on the peristalsis	25 (86.2%)	3 (10,3%)	1 (3.5%)
In nurses' opinions (*n* = 71) massage may			
Improve the sleep quality	58 (81.7%)	11 (15.5%)	2 (2.8%)
Reduce the level of fear and anxiety	53 (74.7%)	12 (16.9%)	6 (8.4%)
Reduce pain	65 (91.6%)	6 (8.4%)	—
Have a positive effect on the peristalsis	64 (90.2%)	6 (8.4%)	1 (1.4%)

**Figure 1:**
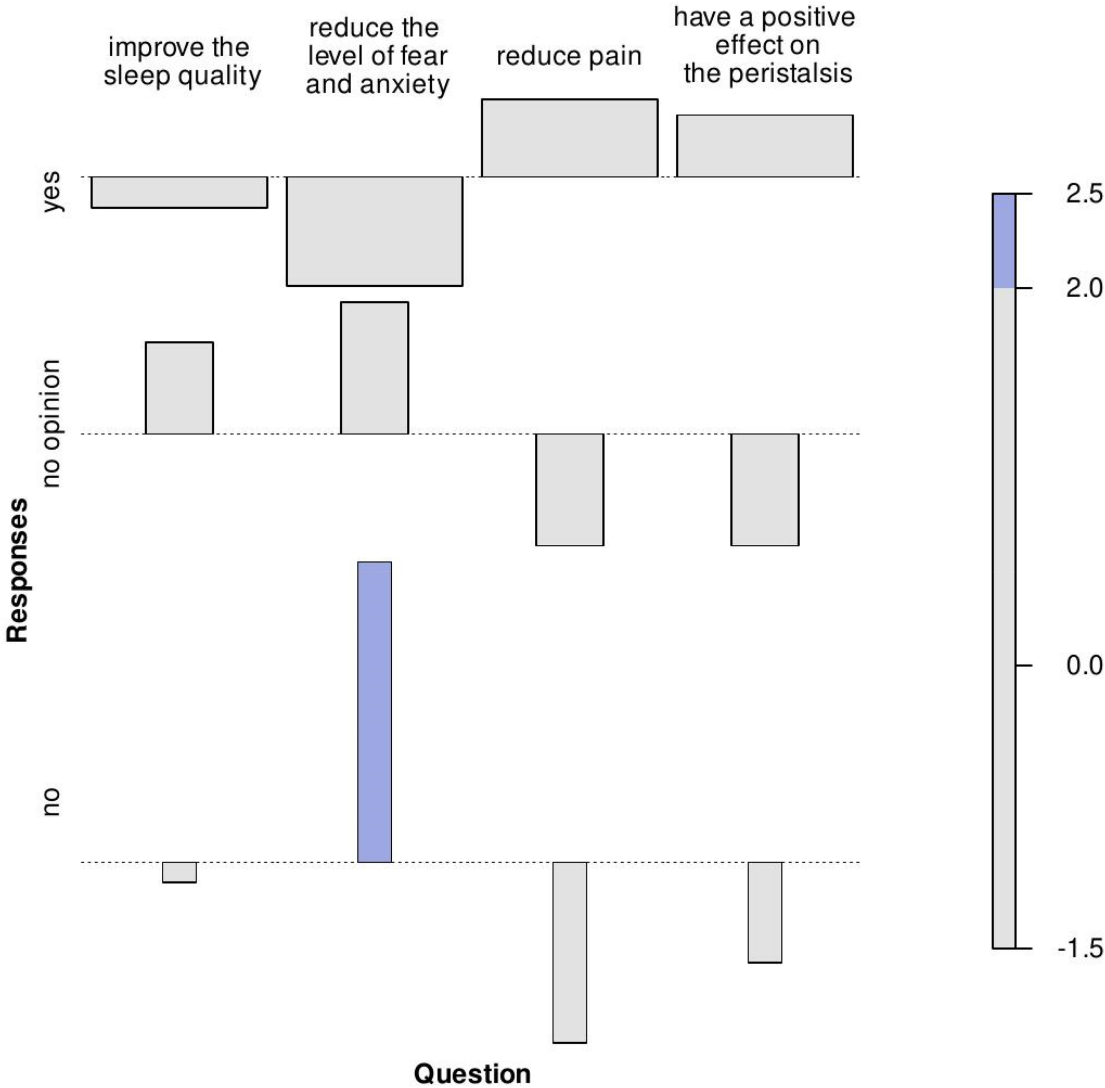
Pearson residuals determined for the contingency table formed on the basis of nurses' opinions expressed on the effect of massage on selected medical problems. In an association diagram, each cell is represented by a rectangle whose area is proportional to the difference in observed and expected frequencies. The rectangles in each row are positioned relative to a baseline indicating independence. If the observed frequency of the cell is higher than expected, the rectangle is above the baseline, otherwise it is below.

It was also investigated whether, in the groups of physiotherapists, physicians and nurses, opinions on the usefulness of massage vary according to the medical problems selected. The Fisher test was used to resolve this question. A non-significant statistical result was obtained for physiotherapists and physicians (*p*-value = 0.63743 and *p*-value = 0.69314 respectively). This means that the usefulness of massage in each of the analyzed medical problems was evaluated similarly. For the professional group of nurses, there are statistically significant differences in the evaluation of the effect of massage on selected medical problems. The result of Fisher's test was statistically significant (*p*-value = 0.03600, pwr = 0.79). The last significantly statistical result was also presented in the form of an association diagram (Figure 1).

Opinions on the usefulness of massage by physical therapists in improving sleep quality among the studied professional groups in the independence test are not statistically significant (*p*-value = 0.93584). The same non-statistically significant results were obtained for the reduction of fear and anxiety (*p*-value = 0.22528), reduce pain (*p*-value = 0.43520) and have a positive effect on the peristalsis (*p*-value = 0.29438). The results mean that physiotherapists, physicians, as well as nurses have a similar (positive) opinion of massage performed by physiotherapists in the prevention and treatment of selected medical problems in ICU patients.

Given the high percentage of positive opinions on the use of massage in ICU patients, one would expect that representatives of the individual health professions would react equally positively to the suggestion of massage not only by physiotherapists, but also by physicians and nurses. The distribution of responses to questions from the second group within each professional group is presented in Table 3 (physiotherapists' opinions), Table 4 (physicians' opinions), and Table 5 (nurses' opinions).

**Table 3:** Physiotherapists' opinions (*n* = 35) on the use of massage in ICU patients.

Question (summarised)	Responses		
	Yes	No opinion	No
	*n* (%)	*n* (%)	*n* (%)
Use of massage by physiotherapists			
Should be a permanent element of the rehabilitation	25 (71.4%)	4 (11.4%)	6 (17.2%)
Is safe	25 (71.4%)	8 (22.9%)	2 (5.7%)
Elements of massage used by ICU physicians			
Could be a part of the basic rehabilitation	7 (20%)	20 (57.1%)	8 (22.9%)
Is safe	7 (20%)	22 (62.9%)	6 (17.1%)
Elements of massage used by ICU nursing staff			
Could be a part of the basic rehabilitation	16 (45.7%)	11 (31,4%)	8 (22.9%)
Is safe	10 (28.6%)	19 (54.3%)	6 (17.1%)

**Table 4:** Physicians' opinions (*n* = 29) on the use of massage in ICU patients.

Question (summarised)	Responses		
	Yes	No opinion	No
	*n* (%)	*n* (%)	*n* (%)
Use of massage by physiotherapists			
Should be a permanent element of the rehabilitation	28 (96.6%)	1 (3.4%)	—
Is safe	25 (86.2%)	3 (10.3%)	1 (3.5%)
Elements of massage used by ICU physicians			
Could be a part of the basic rehabilitation	13 (44.8%)	5 (17.3%)	11 (37.9%)
Is safe	20 (69%)	3 (10.3%)	6 (20.7%)
Elements of massage used by ICU nursing staff			
Could be a part of the basic rehabilitation	16 (55.2%)	5 (17.2%)	8 (27.6%)
Is safe	20 (69%)	7 (24.1%)	2 (6.9%)

**Table 5:** Nurses' opinions (*n* = 71) on the use of massage in ICU patients.

Question (summarised)	Responses		
	Yes	No opinion	No
	*n* (%)	*n* (%)	*n* (%)
Use of massage by physiotherapists			
Should be a permanent element of the rehabilitation	65 (91.6%)	5 (7.0%)	1 (1.4%)
Is safe	63 (88.7%)	8 (11.3%)	—
Elements of massage used by ICU physicians			
Could be a part of the basic rehabilitation	31 (43.7%)	25 (35.2%)	15 (21.1%)
Is safe	30 (42.3%)	25 (35.2%)	16 (22.5%)
Elements of massage used by ICU nursing staff			
Could be a part of the basic rehabilitation	26 (36.6%)	17 (24%)	28 (39.4%)
Is safe	28 (39.4%)	24 (33.8%)	19 (26.8%)

The *χ*2 test and Fisher's test were used for the following six questions in which three professional groups were asked their opinions on the use of massage in the rehabilitation of ICU patients by physiotherapists, doctors and nurses. For the question: “The use of massage by physiotherapists should be a permanent element in the rehabilitation of ICU patients,” the result of Fisher's test was significantly statistical (*p*-value = 0.00609, pwr = 0.85) as was the result of *χ*2 test (*p*-value = 0.00980, pwr = 0.87) for the question: “The elements of massage applied by ICU doctors, after appropriate training, can be part of the basic rehabilitation procedures of ICU patients.” In contrast, the result of the *χ*2 test was not significantly statistically significant (*p*-value = 0.25645) for the question: “Massage elements used by ICU nursing staff can be part of the basic rehabilitation procedures of ICU patients.” The lack of statistical significance means that the opinion on the question asked is similar in each professional group. In the case of the question: “The use of massage by physiotherapists is safe for ICU patients.” The result of Fisher's test was not statistically significant (*p*-value = 0.08655). The *χ*2 test results for the following two questions: “Massage elements used by ICU physicians, with appropriate training, would be safe for ICU patients.” and “Massage elements used by ICU nursing staff would be safe for ICU patients.” were significantly statistically significant (*p*-value = 0.00028, pwr = 0.98 and *p*-value = 0.00465, pwr = 0.89). Statistical significance in this case means that the opinions on the questions asked are not similar across the professional groups studied. Taking into account the respondents' opinions about the application of massage by physiotherapists as a permanent part of the rehabilitation process of ICU patients there is a general consensus among all the professional groups participating in the survey (Tables 3–5). A noteworthy fact is that PTs (Table 3) were the professional group that gave the least positive answers to this question, with 71.4% (25/35), compared to PHs (Table 5) with 96.6% (28/29) and RNs (Table 5) with 91.6% (65/71). However, when it came to using of massage as part of the rehabilitation process by PHs, only 20% (7/35) of PTs approved of this possibility (Table 3). In contrast, more than twice as many physiotherapists, 45.7% (16/35), would approve of the possibility of massage applied by nursing staff during the basic rehabilitation procedures of ICU patients (Table 3). It is interesting to note that as many as 44.8% (13/29) of PHs (Table 4) perceived that physicians could perform massage during basic rehabilitation procedures for ICU patients, and only 36.6% (26/71) of RNs (Table 5) regard the possibility for their professional group to apply massage elements on the aforementioned group of patients.

Another issue presented in Tables 3–5 was the safety of the use of massage by PTs, RNs and PHs working in the intensive care unit. It is worth noting that when physiotherapists assessed the potential safety of massage performed by PHs and RNs (Table 3), they most often evaded answering both “no opinion” on PHs 62.9% (22/35) and on RNs 54.3% (19/35). While 86.2% (25/29) PHs (Table 4) and 88.7% (63/71) RNs (Table 5) considered massage performed in intensive care units by PTs to be the safe form of intervention. Admittedly, 69% (20/29) PHs (Table 4) and 39.4 (28/71) RNs (Table 5) perceived massage as a safe procedure if it were to be performed by themselves. However, it is important to notice that as many as 20.7% of PHs (Table 4) considered that the massage performed by PHs on ICU patients would be dangerous. Similarly, 26.8% of nurses (Table 5) stated that massage performed by nurses would not be a safe procedure for ICU patients. Also, in the opinion of PTs massage would not be safe when performed by PHs and RNs which is shown in Table 3.

## 4 Discussion

The current study shows that massage is perceived by medical staff working in ICUs as a safe treatment for patients treated in this way and can be a permanent element of the physiotherapy process especially when performed by physiotherapists. When comparing the opinions of the three professional groups, no statistically significant difference was obtained (*p* = 0.08655), indicating that they have similar opinions on the subject. When applying massage during physiotherapy of ICU patients the indications and contraindications for this form of therapy and specific conditions (type of procedure, injury, presence of vascular access etc) should also be considered. Physiotherapists (PTs), physicians (PHs), as well as registered nurses (RNs) practicing in the ICU unanimously regard massage, when performing by PTs, as a beneficial part of the rehabilitation process that can improve the quality of sleep and bowel movements and reduce pain, anxiety and distress in ICU patients. However, the undisputed therapeutic value of using massage with patients in the ICU does not translate into everyday practice. The main barrier to physiotherapy treatments, including massage, is the limited number of physiotherapists employed in the ICU (20). It is possible that, for this reason, there was no homogeneity among the clinicians surveyed on the use of massage as a regular part of physiotherapy procedures (*p* = 0.006090). The observed barriers reflect broader organizational and systemic challenges in healthcare delivery, including staff shortages, role ambiguity, and limited interdisciplinary collaboration. The disproportionate reliance on PTs to perform massage underscores the need for a systematic approach that includes role clarification, interdisciplinary training, and equitable workload distribution to optimize resource utilization and uphold the principles of equity in healthcare.

The reasons why PHs and RNs are insufficiently involved in the rehabilitation of ICU patients have already been studied. These have primarily focused on identifying barriers that non-physiotherapists, mainly nursing staff, face in their rehabilitation work with ICU patients and on the early mobilization procedure and its implementation (21). These barriers can be divided into four categories: related to the clinical condition of the patient (stable/unstable, relative/absolute contraindications to mobilization, etc.); structural—including human and technical resources (e.g., availability of staff, appropriate equipment, protocols); cultural (customs, attitudes); related to the process, including the way of providing services (including the division of roles and responsibilities). However, from the point of view of the removal of individual types of barriers, they are divided into: modifiable (through the implementation of appropriate strategies) and non-modifiable.

According to available data, the most common barrier related to the patient's clinical condition is hemodynamic instability, the presence of vascular access devices, drains, etc., deep sedation or decreased level of consciousness, pain, fatigue, drowsiness, refusal to cooperate, etc. In the case of this group, the proposed strategies are: interdisciplinary approach to improve the early mobilization procedure, development and implementation of appropriate protocols and definition of criteria for inclusion and exclusion of the patient from therapy. In terms of structural limitations, the most common problem is insufficient staff or their inadequate training and the lack of an organized program for early mobilization of the patient. They are also associated with cultural barriers related to EM, where early mobilization is not *a priority*, there is a lack of interdisciplinary cooperation in this area, and there is reluctance to conduct procedures resulting most often from insufficient knowledge and experience. In terms of the method of providing services, limitations are related to the organization of the process—lack of planning and coordination, unclear roles and responsibilities of individual members of the therapeutic team, lack or delay of control tests affecting qualification for rehabilitation. Cultural and organizational barriers can be removed by implementing activities including education (e.g., theoretical and practical training, instructional films, etc.), interdisciplinary cooperation in decision-making and setting goals, division of roles and responsibilities, greater involvement of staff and the patient's family, timely provision of information/test results enabling the initiation of EM, but also feedback on the achievement of subsequent stages of mobilization by the patient (22–26). For example, the implementation of evidence-based frameworks like the ABCDEF bundle could provide a structured pathway for integrating massage into ICU care. This framework promotes collaboration across professional groups while addressing key patient care dimensions, including pain management and early mobility.

Considering both the physical and mental consequences of critical illness and the lack of clear protocols for the management of patients in ICU, in 2013 the Society of Critical Care Medicine (SCCM) proposed such a standard aimed at increasing the chances of recovery and minimizing unfavorable factors, called the ABCD bundle. The PAD package—pain, agitation, delirium proved to be insufficient and in 2018 it was expanded to include early mobilization and family engagement and empowerment. The new package—ABCDEF helps in organizing patient care within a multidisciplinary team, including optimal use of resources. It results in improved patient cooperation, the possibility of safe participation in physical exercises, and helps prevent cognitive decline, post-traumatic stress disorder or depression. The ABCDEF package includes: Assess, Prevent, and Manage Pain, Both Spontaneous Awakening Trials (SAT) and Spontaneous Breathing Trials (SBT), Choice of analgesia and sedation, Delirium: Assess, Prevent, and Manage, Early Mobility and Exercise, and Family Engagement and Empowerment (27).

The management of pain, in addition to standard pharmacotherapy, non-pharmacological procedures are increasingly included, such as massage, music therapy, acupuncture, etc. (6, 28–29). Massage therapy is reported to lessen anxiety and decrease postoperative pain among patients in ICU, also the hand and foot massage is reported as an effective way to reduce pain (4, 30–33). Combination of massage and music therapy has proven more effective than each of this technique alone. Moreover, not only in pain reduction, but also improves the vital signs in ICU patients (34, 35).

Liew et al. described barriers to early mobilisation used by 13 nurses in a Singapore hospital (36). Time constraints, safety concerns, and patient resistance were noted. Facilitators included hands-on training, teamwork, and positive outcomes. Zhang et al. collected data from 485 nurses working in 188 hospitals in China's Guizhou province during a nearly three-year study (37). Significant positive correlations between knowledge and practice, and attitude and practice were identified. Similarly, among physiotherapists, significant barriers to early mobilization have been observed (38). The obstacles included lack of professional autonomy, motivation, and clinical skills.

Likewise, the implementation of early mobilization conducted in two or more professional groups has been the subject of studies by other researchers. For instance, Lewis et al. compared the experiences of nursing staff and physiotherapists (135 in total) from a single institution (39). While Lin et al. conducted a survey among physiotherapists, physicians and nurses from a single ICU unit of an Australian hospital, and the study group included 82 respondents (40). In three Montreal hospitals, a survey among physiotherapists, physicians and nurses was conducted by Anekwe et al. in 2019, the results were published on a group of 138 ICU staff (41), and in 2020 on a group of 33 (42). In Rome, on the other hand, Salvitti et al. conducted a study on a group of 29 representatives of three professional groups (20). The variety of ways in which the topic is covered means that only a few articles can qualify for a systematic literature review (21). It seems that massage as one of the elements of work with ICU patients has not yet been the subject of analyses of the possibility of involving professional groups other than physiotherapists. There are significant similarities between the two elements of therapy—early mobilization and massage. Both are equally simple to carry out and yet are extremely important for improving the patient's condition. For this reason, the current study specifically refers to the work on early mobilization in the discussion.

The gap between high levels of knowledge about massage benefits and the reluctance to incorporate it into practice reveals an organizational barrier. Training and education could bridge this gap, enabling interdisciplinary contributions (43). To sum up the analysis of the scientific reports on the subject, researchers have defined three groups of barriers to rehabilitation tasks (21, 39). These are organizational, individual and patient-related barriers.

In the current study the affirmative answers to the questions about the effectiveness of massage in selected medical problems (Table 1) are considered to be based on knowledge and familiarity with the literature and clinical experience on the subject. From this point of view, the current research confirms a good or high level of respondents' knowledge. In the context of the findings of some of the authors, this is not at all obvious. Insufficient levels of knowledge have been defined as one of the barriers to introducing early mobilization into ICU clinical practice (41, 42). In the case of the Montreal study, half of the respondents do not consider early mobilization to be an important thing in the treatment of ICU patients. The researchers believe that this position is due to insufficient knowledge (41). The current study describes a different situation depending on the professional group, in which a high level of knowledge does not translate into a willingness to carry out additional) tasks. As expected, 71.4% (25/35) of physiotherapists consider that massage should be a permanent part of the physiotherapy of the ICU patient (Table 3). On the other hand, it is questionable why only 36.6% (26/71) of the nurses taking part in the survey agreed that massage could be performed by their professional group during elementary rehabilitation procedures (Table 5). On the other hand, it seems rather unexpected that 44.8% (13/29) of physicians see the possibility for their professional group to perform massage as part of elementary rehabilitation procedures (Table 4). It is worth noting that only 20% (7/35) of physiotherapists would see the possibility for physicians to perform any element of massage on ICU patients (Table 3), while as many as 43.7% (31/71) of nurses would trust physicians and consider it possible for physicians to massage critically ill patients (Table 5).

The gap between knowledge and readiness to act, evident in the results of the current study, should be categorized as an organizational barrier. In a study by Zhang et al. on nurses' attitudes, knowledge was shown to translate into the performance of specific rehabilitation activities (37). In contrast, the results of the presented survey indicate that as many as 39.4% of nurses (28/71) would not consider that their professional group could perform massage as part of the ICU patients' basic rehabilitation procedures (Table 5). A study by Liew et al. shows that nurses who declare a willingness to engage in physiotherapy tasks point out that representatives of other professional groups do not offer support (36). At the same time, it is emphasized in the discussion of the results of the South African and Zimbabwean studies that physiotherapists cannot work without the support of other persons among ICU staff (38). The current study describes a situation of all groups of medical professionals in the ICU possibly involved in performing massage among ICU patients.

The current research allows arguing that, in addition to knowledge and skills, the ICU workload factor is equally important. It is identical to the lack of time to perform early mobilization tasks indicated in the literature (36, 39, 40, 42). It is partly related to the situation of a shortage or an inadequate number of physiotherapists (20). It seems that it may be challenging to implement physiotherapists into an additional rehabilitation procedure such as massage, despite the potential benefits for ICU patients. Furthermore, the involvement of physicians and nurses working in the ICU to perform massage is also very difficult to implement in reality, based on the research presented and the scientific studies reported.

## 5 Limitations

A limitation of our study is that the forms of employment of physiotherapists vary between the centers included in the study. It is in Kraków only, where care is provided to patients by a physiotherapist 24 h a day and only in this centre physiotherapists are employed on a fulltime basis. In Koscierzyna, this care is casual. Not every centre was able to obtain responses from representatives of all three professional groups, e.g., in Krakow, only physiotherapists and nurses completed the questionnaires. This limited geographic and institutional scope may affect the generalizability of the results, as the perceptions of clinicians from different regions or healthcare systems might differ. Lastly, the study did not examine the actual clinical outcomes of using massage in ICU rehabilitation but rather focused on the perceptions of the healthcare staff. Additionally, the study did not explore the cultural and contextual factors influencing massage adoption in ICUs, such as institutional attitudes toward non-pharmacological interventions or patient preferences. Incorporating these factors in future research could offer a more comprehensive understanding of the barriers and facilitators to massage implementation in ICU settings. Future studies would benefit from incorporating clinical trials or observational studies to directly measure the effects of massage on patient outcomes such as pain, anxiety, and recovery rates.

## 6 Conclusions

According to all healthcare professionals involved in the study: physiotherapists, nurses and physicians, massage should be a permanent part of the physiotherapy process in the ICU, as a safe and useful part of the rehabilitation process. Massage is perceived to have a positive effect on improving sleep quality and bowel movements and also on reducing anxiety, restlessness and pain in ICU patients. Medical staff working in the ICU recommend that massage is mainly performed by physiotherapists working in ICU, as this ensures that this therapy is carried out professionally and safely. However, further research is needed on the safety and exact methodology of performing massage on ICU patients. In addition, the use of massage among ICU patients should be reviewed in terms of a particular ICU, cultural differences, and current medical law.

## 7 Clinical implications

The findings suggest that massage should be integrated as a regular component of the rehabilitation process for ICU patients, particularly when performed by trained physiotherapists. However, as there is a worldwide shortage of medical staff working in ICUs (20, 21, 38, 39, 42, 44), there is a barrier to the implementation of massage by physiotherapists. Therefore, consideration should be given to involving the patient's family and relatives in performing the basic elements of massage after appropriate training by an experienced physiotherapist. Such procedures have already been applied in some hospitals to the benefit of ICU patients (3).

## Data Availability

The raw data supporting the conclusions of this article will be made available by the authors, without undue reservation.
