# Supplementary material for: Clinicians' opinion on massage in the intensive care unit patients
Source: Front Pain Res (Lausanne). 2025 Apr 9;6:1452434. doi: 10.3389/fpain.2025.1452434 (PMC12014692; doi:10.3389/fpain.2025.1452434)
Supplement: Supplementary file 1 [file Datasheet1.pdf]

Table 6. Checklist for Reporting Results of Internet E-Surveys (CHERRIES)

Item category	***Checklist Item***	***Explanation***
Describe survey design	Describe target population, sample frame. Is the sample a convenience sample? (In “open” surveys this is most likely.)	medical personnel working in ICUs in three medical institutions
IRB approval	Mention whether the study has been approved by an IRB.	The Independent Bioethics Committee for Research at the Medical University of Gdansk, after reviewing the preliminary draft of this survey, has stated in an official email response that the survey presented does not require official approval.
Informed consent	Describe the informed consent process. Where were the participants told the length of time of the survey, which data were stored and where and for how long, who the investigator was, and the purpose of the study?	Participants read the Participant Disclosure Form, but it did not require a signature due to the anonymous nature of the survey. In the Participant Disclosure Form was the aim of the study and the length of time to complete the survey.
Data protection	If any personal information was collected or stored, describe what mechanisms were used to protect unauthorized access.	The data was protected by personal password needed to get an access to the computer and the Google Form of the researcher.
Development and testing	State how the survey was developed, including whether the usability and technical functionality of the electronic questionnaire had been tested before fielding the questionnaire.	The survey was developed based on the current literature and the practical experience of the researcher.
Open survey versus closed survey	An “open survey” is a survey open for each visitor of a site, while a closed survey is only open to a sample which the investigator knows (password-protected survey).	The closed survey when questionnaires were delivered in paper form to each staff member of the hospital wards surveyed in person. Also the closed survey when delivered online to selected groups of the particular ICU staff.
Contact mode	Indicate whether or not the initial contact with the potential participants was made on the Internet. (Investigators may also send out questionnaires by mail and allow for Web-based data entry.)	Initial contact was made in person by the physiotherapists employed in the intensive care units.
Advertising the survey	How/where was the survey announced or advertised? Some examples are offline media (newspapers), or online (mailing lists – If yes, which ones?) or banner ads (Where were these banner ads posted and what did they look like?). It is important to know the wording of the announcement as it will heavily	The survey was announced in person through interviews with staff, coordinators and heads of intensive care units.

	influence who chooses to participate. Ideally the survey announcement should be published as an appendix.	
Web/E-mail	State the type of e-survey (eg, one posted on a Web site, or one sent out through e-mail). If it is an e-mail survey, were the responses entered manually into a database, or was there an automatic method for capturing responses?	Questionnaires were distributed and collected both online (directly by respondents to Google Form system) and in written form by physiotherapists working in ICUs. The written questionnaires were manually loaded into the Google Form system. Next, the number and type of the participants were downloaded from the Google Forms system directly. Then, the final data set was exported into Microsoft Excel spreadsheet
Context	Describe the Web site (for mailing list/newsgroup) in which the survey was posted. What is the Web site about, who is visiting it, what are visitors normally looking for? Discuss to what degree the content of the Web site could pre-select the sample or influence the results. For example, a survey about vaccination on a anti-immunization Web site will have different results from a Web survey conducted on a government Web site	The survey was not made publicly available online. It was distributed in person to selected recipients, i.e. clinicians from the surveyed ICUs (either electronically or on paper).
Mandatory/voluntary	Was it a mandatory survey to be filled in by every visitor who wanted to enter the Web site, or was it a voluntary survey?	voluntary
Incentives	Were any incentives offered (eg, monetary, prizes, or non-monetary incentives such as an offer to provide the survey results)?	none
Time/Date	In what timeframe were the data collected?	January 2021 – June 2022
Randomization of items or questionnaires	To prevent biases items can be randomized or alternated.	The order of survey questions was the same for each participant.
Adaptive questioning	Use adaptive questioning (certain items, or only conditionally displayed based on responses to other items) to reduce number and complexity of the questions.	N/A
Number of Items	What was the number of questionnaire items per page? The number of items is an important factor for the completion rate.	4 questions about participant and 10 questions on the topic of research – up to 11 questions per page
Number of screens (pages)	Over how many pages was the questionnaire distributed? The number of items is an important factor for the completion rate.	3

Completeness check	It is technically possible to do consistency or completeness checks before the questionnaire is submitted. Was this done, and if “yes”, how (usually JAVAScript)? An alternative is to check for completeness after the questionnaire has been submitted (and highlight mandatory items). If this has been done, it should be reported. All items should provide a non-response option such as “not applicable” or “rather not say”, and selection of one response option should be enforced.	Yes it is, utilizing Google Forms functionalities, non-response options percentage varied from question to question, from 0% to 37%
Review step	State whether respondents were able to review and change their answers (eg, through a Back button or a Review step which displays a summary of the responses and asks the respondents if they are correct).	Not used.
Unique site visitor	If you provide view rates or participation rates, you need to define how you determined a unique visitor. There are different techniques available, based on IP addresses or cookies or both.	Not used.
View rate (Ratio of unique survey visitors/unique site visitors)	Requires counting unique visitors to the first page of the survey, divided by the number of unique site visitors (not page views!). It is not unusual to have view rates of less than 0.1 % if the survey is voluntary.	Not used.
Participation rate (Ratio of unique visitors who agreed to participate/unique first survey page visitors)	Count the unique number of people who filled in the first survey page (or agreed to participate, for example by checking a checkbox), divided by visitors who visit the first page of the survey (or the informed consents page, if present). This can also be called “recruitment” rate.	In the case of this survey, we report ‘survey return’. This is due to the fact that some surveys were completed on paper and some were completed electronically. There were 266 people asked, mainly through the physiotherapists working on the ward, but also through the coordinators of the medical, nursing and physiotherapy staff. The survey was eventually completed by 135 people, meaning that 51 per cent of those who were asked to complete the survey did so.
Completion rate (Ratio of users who finished the survey/users who	The number of people submitting the last questionnaire page, divided by the number of people who agreed to participate (or submitted the first survey page). This is only relevant if there is a separate “informed consent” page or if the survey goes over several pages. This is a measure for attrition. Note that	Please see response above.

agreed to participate)	“completion” can involve leaving questionnaire items blank. This is not a measure for how completely questionnaires were filled in. (If you need a measure for this, use the word “completeness rate”.)	
Cookies used	Indicate whether cookies were used to assign a unique user identifier to each client computer. If so, mention the page on which the cookie was set and read, and how long the cookie was valid. Were duplicate entries avoided by preventing users access to the survey twice; or were duplicate database entries having the same user ID eliminated before analysis? In the latter case, which entries were kept for analysis (eg, the first entry or the most recent)?	No.
IP check	Indicate whether the IP address of the client computer was used to identify potential duplicate entries from the same user. If so, mention the period of time for which no two entries from the same IP address were allowed (eg, 24 hours). Were duplicate entries avoided by preventing users with the same IP address access to the survey twice; or were duplicate database entries having the same IP address within a given period of time eliminated before analysis? If the latter, which entries were kept for analysis (eg, the first entry or the most recent)?	Not verified.
Log file analysis	Indicate whether other techniques to analyze the log file for identification of multiple entries were used. If so, please describe.	Not verified.
Registration	In “closed” (non-open) surveys, users need to login first and it is easier to prevent duplicate entries from the same user. Describe how this was done. For example, was the survey never displayed a second time once the user had filled it in, or was the username stored together with the survey results and later eliminated? If the latter, which entries were kept for analysis (eg, the first entry or the most recent)?	Not applied.

Handling of incomplete questionnaires	Were only completed questionnaires analyzed? Were questionnaires which terminated early (where, for example, users did not go through all questionnaire pages) also analyzed?	No.
Questionnaires submitted with an atypical timestamp	Some investigators may measure the time people needed to fill in a questionnaire and exclude questionnaires that were submitted too soon. Specify the timeframe that was used as a cut-off point, and describe how this point was determined.	Not applied.
Statistical correction	Indicate whether any methods such as weighting of items or propensity scores have been used to adjust for the non-representative sample; if so, please describe the methods.	No.

This checklist has been modified from Eysenbach G. Improving the quality of Web surveys: the Checklist for Reporting Results of Internet E-Surveys (CHERRIES). J Med Internet Res. 2004 Sep 29;6(3):e34 [erratum in J Med Internet Res.2012; 14(1): e8.]. Article available at https://www.jmir.org/2004/3/e34/; erratum available https://www.jmir.org/2012/1/e8/. Copyright ©Gunther Eysenbach. Originally published in the Journal of Medical Internet Research, 29.9.2004 and04.01.2012.

This is an open-access article distributed under the terms of the Creative Commons Attribution License (https://creativecommons.org/licenses/by/2.0/), which permits unrestricted use, distribution, and reproduction in any medium, provided the original work, first published in the Journal of Medical Internet Research, is properly cited.
