# Supplementary material for: Clinicians' opinion on massage in the intensive care unit patients
Source: Front Pain Res (Lausanne). 2025 Apr 9;6:1452434. doi: 10.3389/fpain.2025.1452434 (PMC12014692; doi:10.3389/fpain.2025.1452434)
Supplement: Supplementary file 2 [file Datasheet2.pdf]

Supplementary Material

1 Appendix – questionnaire

Survey: Clinicians' opinion on massage in the Intensive Care Unit patients

~~The feasibility for safe implementation of massage in the intensive care unit~~

Dear Sir or Madame,

We investigate the possibility of using massage in the rehabilitation process of ICU patients.

We kindly request people who work in the ICU to fill in this questionnaire.

The survey is completely anonymous and will take approximately 5 minutes to complete.

Work experience in ICU in the years: ………..

Please circle the answer.

Sex (select the correct one): male / female

Occupation (select the appropriate one): Doctor / Nurse / Physiotherapist

PART ONE – the use of massage by ICU physiotherapists

1. The use of massage by physiotherapists should be a permanent element of the rehabilitation of ICU patients.

Yes No No opinion

2. The use of massage by physiotherapists is safe for ICU patients.

Yes No No opinion

3. The use of massage by physiotherapists may have a positive effect on the peristalsis of the large intestine and the prevention of constipation in ICU patients.

Yes No No opinion

4. The use of massage by physiotherapists may improve the sleep quality of ICU patients.

Yes No No opinion

5. The use of massage by physiotherapists may reduce the level of fear and anxiety in ICU patients.

Yes No No opinion

6. The use of massage by physiotherapists may reduce pain in ICU patients.

Yes No No opinion

PART TWO – the use of massage by ICU nursing staff

1. Elements of massage used by ICU nursing staff could be a part of the basic rehabilitation procedures of ICU patients.

Yes No No opinion

2. Elements of massage used by ICU nursing staff would be safe for ICU patients.

Yes No No opinion

PART THREE – the use of massage by ICU doctors.

1. Elements of massage used by ICU doctors, after appropriate training, could be a part of the basic rehabilitation procedures of ICU patients.

Yes No No opinion

2. Elements of massage used by ICU doctors, after appropriate training, would be safe for ICU patients.

Yes No No opinion

Thank you very much for your time
